# Cardiac strangulation with chronic ab-extrinseco occlusion of the left-circumflex artery from an epicardial lead: a case report

**DOI:** 10.1093/ehjcr/ytae688

**Published:** 2025-01-13

**Authors:** Lorenzo Giarletta, Eleonora Moliterno, Francesco Perna, Riccardo Marano

**Affiliations:** Department of Radiological and Hematological Sciences, Section of Radiology, Università Cattolica del Sacro Cuore, Largo Agostino Gemelli 8 - 00168 Rome, Italy; Department of Radiological and Hematological Sciences, Section of Radiology, Università Cattolica del Sacro Cuore, Largo Agostino Gemelli 8 - 00168 Rome, Italy; Cardiac Arrhythmia Unit, Department of Cardiovascular Sciences, Fondazione Policlinico Universitario Agostino Gemelli IRCCS, Largo Agostino Gemelli 8 - 00168 Rome, Italy; Department of Radiological and Hematological Sciences, Section of Radiology, Università Cattolica del Sacro Cuore, Largo Agostino Gemelli 8 - 00168 Rome, Italy; Cardiovascular Diagnostic Unit, Department of Diagnostic Imaging Oncological Radiotherapy and Hematology, Fondazione Policlinico Universitario Agostino Gemelli IRCCS, Largo Agostino Gemelli 8 - 00168 Rome, Italy

**Keywords:** Cardiac strangulation, Coronary artery occlusion, Epicardial pacemaker, Case report

## Abstract

**Background:**

Cardiac strangulation (CS) from epicardial pacing leads (EPLs) is a rare and potentially lethal mechanical complication associated with epicardial pacemaker (PM) implantation.

**Case summary:**

We report a case of a 44-year-old-female patient presenting with chest and left shoulder pain in the absence of reported trauma with history of congenital atrioventricular block treated with epicardial PM implantation during the childhood and subsequent transvenous reimplantation over the years. Troponin I resulted within normal values and ECG, transthoracic echocardiography and chest X-ray documented no acute cardiopulmonary findings. After 3 months the patient underwent coronary computed tomography angiography (CCTA) documenting the presence of solid and focally calcified tissue grown along the course of the EPLs, determining multiple focal impressions on the left ventricular epicardial edge and a segmental occlusion of the middle left-circumflex artery (LCX) due to ab-extrinseco compression. After 10 days, the patient was admitted at the emergency department with atypical chest pain and underwent invasive coronary angiography (ICA), which confirmed chronic occlusion of the mid-LCX with complete collateral circulation; a stress echocardiography ruled out myocardial ischaemia and the patient was uneventfully discharged.

**Discussion:**

The diagnosis of CS in patients with epicardial PM leads remains challenging, especially in adults with atypical clinical presentation; thus, any clinical or instrumental clue should prompt further higher-level imaging investigations, such as CCTA or ICA. It is also important to disclose that sometimes CS can be only a collateral finding with no relationship with the patient’s symptoms.

**Learning points**

- Cardiac strangulation (CS) is a rare mechanical complication of epicardial pacemaker implantation, possibly leading to coronary artery ‘ab-extrinseco’ stenosis and occlusion, myocardial ischaemia, heart failure and cardiac arrhythmias.
- Remotely implanted abandoned epicardial pacing leads, as well as newly implanted functional leads, can cause CS.
- CS is often a collateral finding in asymptomatic patients or in patients undergoing cardiac coronary tomography angiography to rule out coronary artery disease (CAD) as the cause of chest pain, especially in adult patients with chronically implanted epicardial pacing leads.

## Introduction

Cardiac strangulation (CS) is a rare and potentially life-threatening mechanical complication associated with epicardial pacemaker leads (EPLs) implantation in children.^1,2^ High-level imaging investigations, like coronary computed tomography angiography (CCTA) and invasive coronary angiography (ICA), overcome limitations of traditional imaging modalities in suspicious clinical presentations and allow diagnosis in complex cases.^3^

## Summary figure

**Table:** 

Time	Events
Day 0	A 44-year-old woman with history of congenital atrioventricular-block treated with epicardial pacemaker implantation during the childhood and subsequent transvenous leads reimplantation, complains left shoulder pain, in the absence of recent trauma. Blood tests, ECG, transthoracic echocardiography, and chest X-rays show no acute cardiopulmonary issues.
Day 90	Coronary computed tomography angiography detects solid and focally calcified tissue ingrowth along the course of the epicardial leads, determining multiple focal irregularities on the left ventricular epicardial edge and a segmental occlusion of the mid-tract of the left-circumflex artery due to ‘ab-extrinseco’ compression.
Day 100	Patient is re-admitted to the emergency department because of atypical chest pain. Invasive coronary angiography confirms chronic occlusion of the mid-tract of left-circumflex artery with evidence of a distal complete collateral circulation. Dobutamine stress echocardiography rules out myocardial ischaemia. Patient is discharged in stable clinical and hemodynamic conditions.

## Case presentation

A 44-years-old-female patient was admitted to our emergency department (ED) because of worsening left shoulder pain unresponsive to NSAIDs, without history of recent trauma.

At age two, she underwent pacemaker (PM) implantation with EPLs and abdominal subcutaneous pulse generator to treat congenital atrioventricular (AV) block. At age 17, she had her abdominal generator replaced in the left pectoral region with a transvenous endocardial ventricular lead because of malfunction of the EPLs. At age 25, she received new transvenous endocardial leads with a dual-chamber PM. The patient had no clinical issues over the years and underwent regular clinical follow-ups.

**Figure 1:**
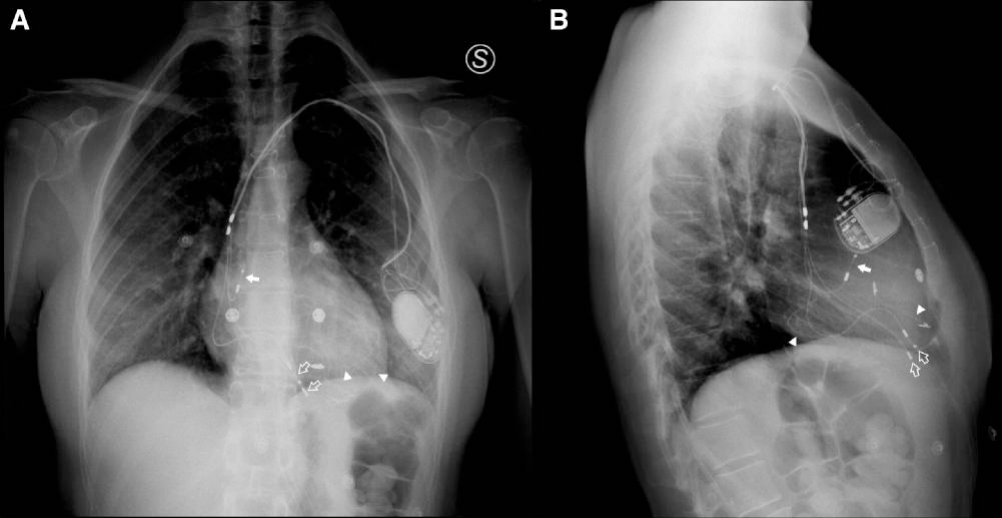
Posterior–anterior (*A*) and lateral (*B*) chest X-rays showing a dual chamber pacemaker generator located in the left antero-lateral chest wall, with the transvenous lead tips located into the right atrial appendage (full arrows) and right ventricular apex (empty arrows), respectively. Multiple abandoned epicardial pacemaker leads along the cardiac surface is also detectable (arrowheads).

On ED admission, she presented with unremarkable cardiovascular physical examination findings and ventricular paced rhythm at ECG, with a sinus heart rate of 85 bmp. Troponin I (two samples 3 h apart), D-dimer and PCR resulted within the normal values. NTproBNP was not significantly elevated (345 pg/mL; normal range <125 pg/mL). Chest X-ray (CXR) documented no acute cardiopulmonary findings (*Figure 1*). Transthoracic echocardiography showed preserved left ventricular (LV) function in the absence of either regional wall motion abnormalities or valvular malfunction. The patient was therefore discharged.

**Figure 2:**
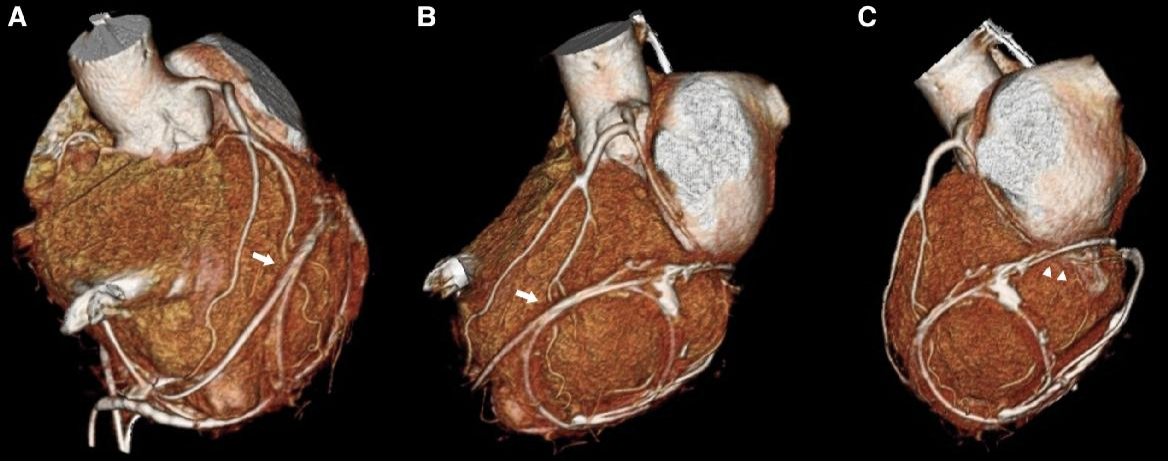
3D volume rendered coronary computed tomography angiography images clearly show multiple epicardial pacemaker leads constricting the apical and posterior-lateral free wall of the left ventricular, crossing the course of the distal first diagonal branch (arrows in *A* and *B*) and the mid-left-circumflex artery (arrowheads in *C*).

**Figure 3:**
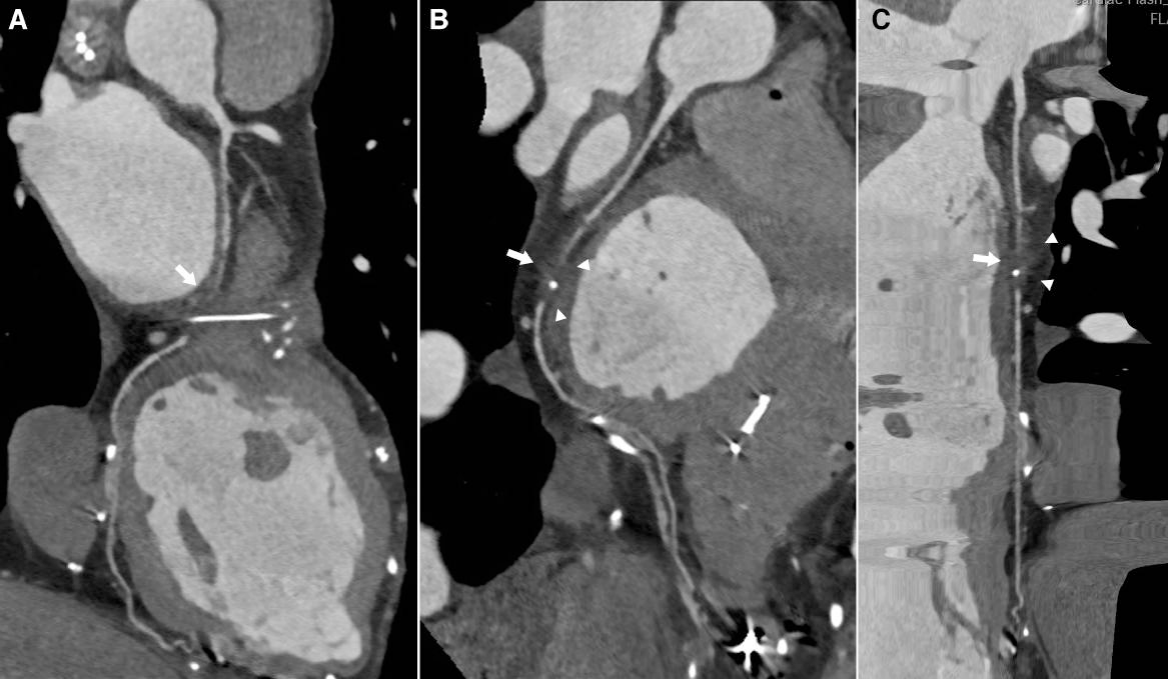
Curved (*A*, *B*) and stretched (*C*) multi-planar coronary computed tomography angiography reconstructions clearly show the segmental occlusion (arrows) of mid-left-circumflex artery due to extrinsic compression by a solid tissue (arrowheads in *B* and *C*) grown along the contiguous and abandoned epicardial lead.

After 3 months, due to drug-refractory recurrent chest and shoulder pain, with negative shoulder echography (MRI was not preferred due to abandoned PM leads), her cardiologist recommended a CCTA to rule out CAD or abnormalities. CCTA was performed in our department revealing solid and focally calcified tissue ingrowth along the course of the EPLs, determining multiple focal irregularities on the LV epicardial border, extended in the middle portion of the left AV groove and resulting in 1.5 cm segmental occlusion of the mid portion of the left-circumflex artery (LCX) due to extrinsic compression. The remaining coronary arteries had no stenosis (*Figures 2* and *3*).

After 10 days, the patient was re-admitted to the ED for atypical chest pain in the last 3 days, worsening on inspiration and left lateral decubitus position. High-sensitivity troponin I was negative in both determinations, as well as PCR, D-dimer, and NTproBNP.

**Figure 4:**
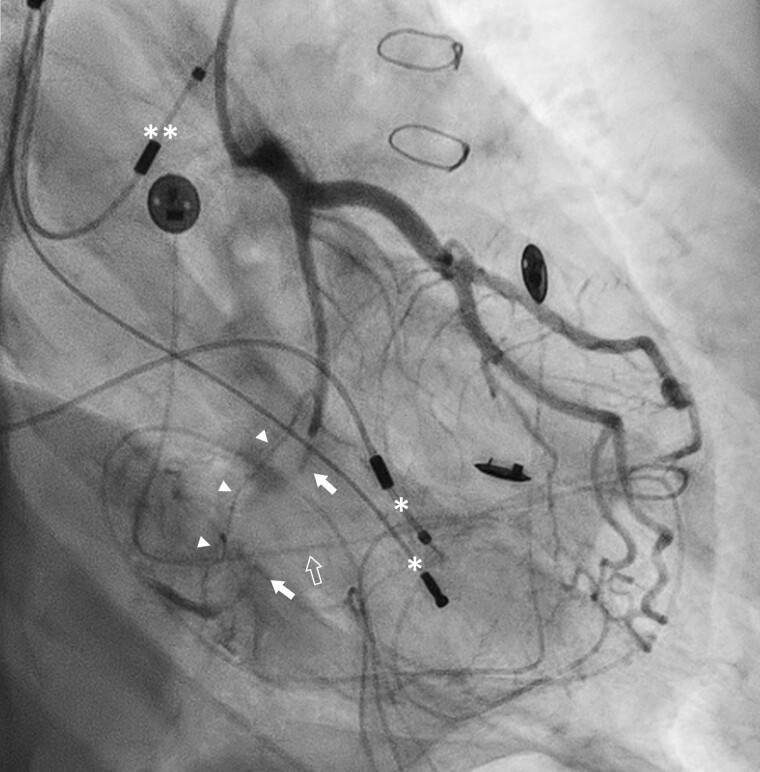
Invasive coronary angiography shows the chronic occlusion of mid-left-circumflex artery (full arrows) at the level of the overlapped epicardial lead (empty arrow), with distal homocoronary collateral circulation (arrowheads). Transvenous right ventricular (*) and atrial (**) leads are shown.

Following the recent CCTA findings, the patient was transferred to the Sub-Intensive Cardiology Department and underwent ICA, which demonstrated left-dominant coronary circulation with chronic total occlusion of the LCX due to ‘ab-extrinseco’ compression from an EPL, with a distal complete homocoronary collateral circulation (*Figure 4*).

A dobutamine stress echocardiography (which was preferred to stress nuclear imaging because of permanent ventricular pacing but preserved sinus node function and to pacing stress test because of our center routine) was performed, showing no inducible myocardial ischaemia, without symptoms elicited. Considering all the investigations performed, we ruled out myocardial ischaemia as the origin of the patient’s symptoms, we reassured her, and she was uneventfully discharged recommending orthopaedical/physiatry consults. After physiotherapy, her symptom progressively improved and disappeared.

## Discussion

CS is a rare yet potentially life-threatening mechanical complication associated with epicardial PM implantation in growing children. It is caused by the progressive compression of the cardiac chambers and great vessels against the previously implanted leads, which are pulled from their anchor points and stretched around the growing heart, possibly leading to ventricular dysfunction, coronary artery extrinsic stenosis, and subsequent myocardial ischaemia, heart failure, and even sudden cardiac death.^1,2^ In fact, like in our case, the EPLs could be pulled towards the coronary arteries during growth and progressively compress them, causing lumen narrowing and even total occlusion. As this condition progress slowly, patients frequently present asymptomatic, like in the current case, when the coronary artery chronic occlusion was incidentally diagnosed in a patient with unrelated symptoms.

Epicardial PM implantation is the standard treatment for congenital AV block in growing children because of the higher complication risk with endocardial leads.^4,5^

In 2015, Carreras *et al*. reviewed their 20-year experience with epicardial PM implantation, noting a 2.3% incidence of CS, and 1.2% mortality related to CS. They described a ‘classic pattern’ of CS that may be observed on CXR, consisting of a posterior ‘heart-shaped’ orientation of the EPLs within the cardiac silhouette on P-A CXR.^1^

In 2018, Mah *et al*.^3^ found a higher incidence (5.5%) of coronary artery compression from EPLs, related to an increased number of publications on the topic, as well as to enhanced CT technology and improved knowledge of the mechanisms of coronary artery compression by EPLs, which may also occur in the absence of CS, without the classical CXR pattern described by Carreras *et al*.^1^ Sometimes EPLs can cause chronic coronary artery occlusion due to the rise of a collateral circulation. It is a challenging diagnosis, especially in asymptomatic adult patients with chronically implanted EPLs.

CXR has high specificity (96%) and negative predictive values (98%), serving as a good screening technique in patients with a low suspicion for CS; however, due to its low sensitivity (57%), Mah *et al*. recommend CCTA or ICA assessment for patients with suggestive or equivocal CXR, concerning symptoms or ventricular dysfunction. CCTA provides specificity and sensitivity of 93% and 100% respectively, while ICA of 100% and 86%, respectively; the latter is also recommended for patients with positive CCTA.^3^

It is noteworthy that extrinsic coronary artery compression and CS can also be caused by abandoned EPLs not connected to any PM.^3,4^ As in our case, fibrous or granulomatous tissue can arise and grow along the EPLs or at the point of attachment on the epicardial surface, adhere to the myocardium and progressively constrict the encompassed structures.^6^

To reduce the risk of CS during epicardial PM implantation procedures, surgeons should minimize the excess lead left within or along the pericardium, although ensuring some redundancy in view of further somatic growth.^7^ CS often depends on the location of the lead’s loop along the anterior epicardial heart surface.^5^ When placing the redundant lead loops, the anterior and apical ventricular surfaces should be avoided, preferring the diaphragmatic surface.^2^ Furthermore, each epicardial electrode should be carefully positioned avoiding proximity to coronary arteries. It has also been suggested that using an expanded polytetrafluoroethylene sheet to separate the heart and leads may prevent CS.^7^

In this case, we found CS to be a serendipitous finding, unrelated to the patient’s clinical scenario. The only way to treat LCX occlusion, if indicated, would have been bypass grafting, which is a highly invasive treatment especially in the presence of a prior sternotomy. Therefore, just as prompt diagnosis and treatment is crucial in high-risk patients, like growing children with EPLs, especially if symptomatic, it is equally important to rule out its involvement when it is only an innocent bystander, to avoid unnecessary invasive interventions.

## Conclusion

CS is a rare mechanical complication of epicardial PM implantation, consisting of progressive heart chambers and great vessels entrapment as well as coronary artery extrinsic compression, or even total occlusion, by EPLs, possibly leading to fatal outcomes. Even abandoned EPLs and the ingrowth of fibrotic and granulomatous tissue along their course may determine coronary artery compression and CS. In patients with previously implanted EPLs, any clinical or CXR suspicion of CS should prompt further higher-level imaging investigations, such as CCTA and ICA.

## Supplementary Material

ytae688_Supplementary_Data

## Data Availability

The data underlying this article are available in the article and in its online Supplementary material.

## Lead author biography

Dr Lorenzo Giarletta is currently a radiology resident at Università Cattolica del Sacro Cuore in Rome, Italy. His clinical and research interests are primarily focused on cardiovascular imaging.

## Notes

**Consent:** The authors confirm that written consent for submission and publication of this case report including images and associated text has been obtained from the patients in line with the Committee on Publication Ethics (COPE) guidelines.

**Funding:** None declared.
